# Refractory Ischemic Priapism Following Hemodialysis

**DOI:** 10.7759/cureus.54185

**Published:** 2024-02-14

**Authors:** Abdalhai Alshoubi, Farris Al-Qawasmi

**Affiliations:** 1 Anesthesiology and Critical Care, OSF Saint Francis Medical Center, Peoria, USA; 2 Anesthesiology, The University of Toledo College of Medicine and Life Sciences, Toledo, USA

**Keywords:** end-stage renal disease, surgical treatment of ischemic priapism, proximal shunts, cavernosal hypoxia, acute ischemic priapism

## Abstract

Priapism, characterized by prolonged and painful penile erection, is a rare urological emergency with diverse etiologies. We present a case of refractory ischemic priapism following hemodialysis in a 57-year-old male with a history of type II diabetes mellitus, hypertension, and end-stage renal disease. Despite standard conservative management, the patient's condition persisted, necessitating penile distal shunting through an intracorporeal dilatation plus Al-Ghorab corporoglandular shunt. Blood gas analysis of corpus cavernosum blood revealed severe acidosis and hypoxemia, emphasizing the systemic impact of ischemic priapism. The patient's history of erythropoietin injections and the administration of heparin during dialysis emerged as potential contributors to priapism. We discuss the complex interplay between erythropoietin, coagulation cascade, and heparin in the context of priapism development. The case underscores the need for further research to understand the specific mechanisms contributing to priapism in patients undergoing hemodialysis.

## Introduction

Priapism is characterized by a prolonged and painful erection of the penis lasting beyond four hours without sexual stimulation. An overall rare condition, priapism accounts for an estimated 1.5 cases per 100,000 in men [1]. Common causes include conditions such as sickle cell disease, trauma, and certain medications [2]. The condition is further classified into three types: ischemic, non-ischemic, and stuttering or recurrent priapism. Ischemic priapism, also known as low-flow priapism, arises from impaired blood outflow from the penis, leading to the entrapment of deoxygenated blood in the corpora cavernosa. Non-ischemic priapism involves increased arterial blood flow, causing abnormal connections or fistulas between arteries and veins. Stuttering priapism is characterized by recurrent ischemic episodes, typically at night, gradually increasing in duration. Among these, ischemic priapism poses the greatest threat due to the lack of oxygen and vital nutrients [3]. It is deemed a urological emergency, necessitating immediate medical attention to prevent potential long-term complications such as erectile dysfunction. Hemodialysis, a common treatment for end-stage renal disease, has been infrequently associated with priapism. The mechanism in the context of hemodialysis is believed to be linked to erythropoietin and inadequate heparinization. While generally considered safe, clinicians should exercise vigilance for potential complications, including priapism, especially in individuals with predisposing factors. Understanding the underlying causes and promptly addressing priapism in the context of hemodialysis are crucial for preventing adverse outcomes and ensuring optimal patient care.

## Case presentation

A 57-year-old male presented to the emergency department with a pertinent medical history including type II diabetes mellitus, hypertension, and end-stage renal disease secondary to diabetic nephropathy. The patient is effectively managed on insulin, daily losartan 50 mg, nifedipine 90 mg, and epoetin alfa 30 mcg injections every three months. Over the past three years, he has undergone home hemodialysis through a left-arm arteriovenous fistula, engaging in this procedure four times a week. The patient's chief complaint was a persistent and painful erection endured for the past 15 hours, originating after a hemodialysis session; heparin was administered during the dialysis session. The patient's initial vital signs upon presentation were within the normal range. Clinical examination revealed an erect and tender penis with rigid corpora cavernosa, whereas the glans and corpora spongiosum exhibited a soft consistency. Aspiration of the corpora cavernosa revealed dark and viscous blood. The patient had no previous incidents of priapism, reported no history of malignancy or hematologic diseases, and denied the use of substances such as cocaine, marijuana, or alcohol. Additionally, he denied any nitrate usage, engagement in sexual intercourse, the use of psychostimulants, as well as the utilization of vasoactive agents or phosphodiesterase inhibitors.

Laboratory analyses showed microcytic anemia, deranged renal function tests, and raised blood sugar levels (Table 1). Further examination of blood from the corpus cavernosum through blood gas analysis revealed hypoxia, hypercapnia, and acidosis (Table 2). A diagnosis of ischemic priapism was established. Despite rigorous attempts at conservative management, which involved multiple corporal aspirations using a 16-G needle and intracavernous injections of 1 mg of epinephrine (250 mcg each time with a five-minute interval), the patient failed to experience detumescence. Subsequently, the decision was made to proceed with penile distal shunting. An intracorporeal dilatation plus Al-Ghorab corporoglandular shunt was performed where a connection was formed between the corpora cavernosa and the glans with dilation of the corpora cavernosa to ensure an effective shunt. This surgical intervention was conducted under general anesthesia, resulting in complete detumescence. The patient was discharged the following day and demonstrated a full recovery. 

**Table 1:** Serum laboratory results on the first and last days of admission WBC: white blood cell; RBC: red blood cell; BUN: blood urea nitrogen; AST: aspartate aminotransferase; ALT: alanine transaminase; INR: international normalized ratio; PT: prothrombin; PTT: partial thromboplastin time

Parameter	Reference range and units	First day of admission	Last day of admission
WBC count	4-10 10^3^/uL	7.2	8.1
RBC count	4.3-5.9 10^6^/uL	2.83	3.14
Hemoglobin	14-18 g/dL	8.8	9.3
Hematocrit	39-49%	26.4	28.2
Mean corpuscular volume	80-99 fL	75.8	85.4
Red cell distribution width	11.4-14.6%	18.5	20.1
Platelet count	150-400 10^3^/uL	230	220
Lymphocytes	16-45%	17	22
Neutrophils relative percent	42-75%	64	62
Monocytes	2-12%	6	7
Eosinophils	0-5%	2	0
Basophils	0-2%	0.3	1
Sodium	135-145 mmol/L	142	139
Potassium	3.5-5.1 mmol/L	4.3	4.2
Chloride	98-107 mmol/L	99	100
Carbon dioxide	21-32 mmol/L	23	25
Glucose	74-106 mg/dL	207	147
BUN	7-18 mg/dL	46	41
Creatinine	0.70-1.30 mg/dL	7.36	3.5
Calcium	8.5-10.1 mg/dL	9.8	10.1
Phosphorus	2.3-4.7 mg/dL	4.1	5.4
AST	15-37 U/L	21	16
ALT	16-61 U/L	23	25
Protein, total	6.4-8.2 gm/dL	5.1	4.1
Albumin	3.4-5 gm/dL	2.8	2.6
Alkaline phosphatase	40-150 U/L	46	52
Bilirubin, total	0.3-1 mg/dL	0.6	2
INR	1	1	1.1
PT	9.4-12.5 seconds	13	14
PTT	25.1-36.5 seconds	32	31
Lactic acid	0.5-2 mmol/L	1.5	1.2

**Table 2:** Cavernous blood gas analysis

	Normal arterial blood	Normal mixed venous blood	Acute ischemic priapism (cavernous blood of our patient)
pH	7.35-7.45	7.35	6.9
pO2 (mmHg)	80-100	40	33
pCO2 (mmHg)	35-45	50	118

## Discussion

Acute ischemic priapism typically exhibits corporal blood gases with a pO2 below 30 mmHg, a pCO2 exceeding 60 mmHg, and a pH below 7.25. In contrast, cavernous blood gases in patients with non-ischemic priapism closely resemble those of arterial blood. The cavernous blood gas levels in the flaccid penis are approximately equivalent to those found in mixed venous blood. The blood gas analysis of the corpus cavernosum blood of our patient demonstrated severe acidosis hypercapnia and hypoxemia, characteristic of ischemic priapism, reinforcing the severity of the condition. The acidic pH observed in the blood gas analysis reflects the presence of accumulated lactic acid and other acidic metabolites, indicating a state of metabolic derangement within the erectile tissue. The combination of hypercapnia, hypoxemia, and acidosis indicated the depletion of all substrates required in adenosine triphosphate (ATP) production. In ischemic priapism, the halt in circulation results in oxygen being consumed with the accompanying production of carbon dioxide and depletion of glucose. The outcome of glycopenia and ATP deficiency will ultimately result in the apoptosis of the spongy tissues within the corpus cavernosa [4]. The glycopenic environment within the cavernosa may likely contribute to the ineffectiveness of phenylephrine and other sympathomimetic drugs [4]. The initiation of priapism following a hemodialysis session prompts consideration of dialysis-related factors. High hematocrits, hypovolemia, and androgen therapy all increase the risk of dialysis-related priapism [5]. It is recommended to withhold androgen therapy when hematocrit is above 25%. Further, the administration of heparin during the previous day's dialysis session, as documented in medical records, introduces a potential link. Heparin-induced priapism remains unclear, but some theories believe it may be due to antibody generation. With administration of heparin, anti-platelet antibodies may develop. Heparin-induced platelet antibodies can result in the aggregation of platelets that will obstruct the flow of blood within the corpus cavernosa leading to low flow seen in ischemic priapism [6]. In some cases, abrupt cessation of heparin may lead to hypercoagulability that causes thrombosis of both the corpus cavernosa and corpus spongiosum [7]. It is unclear whether priapism in this patient may have occurred due to possible heparin antibody formation or insufficient heparinization. Moreover, the patient's history of erythropoietin injections emerges as a potential contributor to the development of priapism. A comparable case involved a 25-year-old man with chronic renal failure who experienced priapism after hemodialysis and the administration of 2000 units of intravenous erythropoietin. Notably, the patient had recurrent incidents of veno-occlusive priapism during previous hemodialysis and erythropoietin treatments. A crucial observation was made when the patient's erythropoietin dosage was reduced from 2000 units to 1500 units, resulting in the resolution of recurrent priapism episodes [8]. This underscores the potential role of erythropoietin in priapism and prompts a deeper exploration of its influence on the coagulation cascade. Various authors have conducted analyses highlighting the impact of erythropoietin on the coagulation cascade, emphasizing its potential to increase platelet aggregation and inhibit fibrinolysis [9]. The intricate interplay between erythropoietin and the coagulation system warrants further investigation to comprehend its implications in priapism development and refine preventive strategies.

## Conclusions

Refractory ischemic priapism following hemodialysis is a rare but potentially devastating complication that requires prompt recognition and management. Initially, conservative management with the use of corporal aspiration to evacuate stagnant blood is favorable, while surgical shunts may provide treatment in refractory cases. In our specific case presented, hemodialysis may have contributed to an increased risk of developing priapism. Additional research is necessary to further understand the specific mechanism that contributes to priapism in this vulnerable demographic.
